# Effectiveness of Medicare cost-sharing elimination for Cancer screening on utilization

**DOI:** 10.1186/s12913-019-4135-9

**Published:** 2019-06-17

**Authors:** Wendy Yi Xu, Thomas M. Wickizer, Jeah Kyoungrae Jung

**Affiliations:** 1 Division of Health Services Management and Policy, College of Public Health, The Ohio State University, Cunz Hall 208, 1841 Neil Avenue, Columbus, OH 43220 USA; 2 Department of Health Policy and Administration, College of Health and Human Development, The Pennsylvania State University, University Park, Pennsylvania, USA

**Keywords:** Affordable care act, Medicare, Cost-sharing elimination, Preventive screening

## Abstract

**Background:**

The Patient Protection and Affordable Care Act (ACA) eliminated the cost-sharing requirement for several preventive cancer screenings. This study examined the cancer screening utilization of mammogram, Pap smear and colonoscopy in Medicare fee-for-service (FFS) under the ACA.

**Methods:**

The primary data were the 2007–2013 Medicare Current Beneficiary Survey linked to FFS claims. The effect of the cost-sharing removal on the probability of receiving a preventive cancer screening test was estimated using a logistic regression, separately for each screening test, adjusting for the complex survey design. The model was also separately estimated for different socioeconomic and race/ethnic groups.

The study sample included beneficiaries with Part B coverage for the entire calendar year, excluding beneficiaries in Medicaid or Medicare Advantage plans. Beneficiaries with a claims-documented or self-reported history of targeted cancers, who were likely to have diagnostic tests or have surveillance screenings were excluded.

The screening measures were constructed separately following Medicare coverage and U.S. Preventive Services Task Force (USPSTF) recommendations. We measured the screening utilization outcome drawing from claims data, as well as using the self-reported survey data.

**Results:**

After the cost-sharing removal policy, we found no statistically significant difference in a beneficiary’s probability of receiving a colonoscopy (transition period: OR = 1.08, 95% CI = 0.90–1.29; post-policy period: OR = 1.08, 95% CI = 0.83–1.42), a mammogram (transition period: OR = 1.03, 95% CI = 0.91–1.17; post-policy period: OR = 1.07, 95% CI = 0.88–1.30), or a biennial Pap smear (transition period: OR = 0.87, 95% CI = 0.69–1.09; post-policy period: OR = 0.72, 95% CI = 0.51–1.03) in claims-based measures following Medicare coverage. Similarly, we found null effects of the policy change on utilization of colonoscopy among enrollees 50–75 years old, biennial mammograms by women 50–74, and triennial Pap smear tests among women 21–65 in claims-based measures according to USPSTF. The findings from survey-based measures were consistent with the estimates from claims-based measures, except that the use of Pap smear declined since 2011. Further, the policy change did not increase utilization in patients with disadvantaged socioeconomic characteristics. Yet the disparate patterns in adjusted screening rates by socioeconomic status and race/ethnicity persisted over time.

**Conclusions:**

Removing out-of-pocket costs for screenings did not provide enough incentives to increase the screening rates among Medicare beneficiaries.

## Background

Cancer is a leading cause of deaths in the United States, being responsible for 22% of all deaths in 2016 [1]. While several commonly diagnosed cancers can be detected earlier by preventive screenings, cancer screening rates in the eligible populations remain below national goals [2]. A large body of literature has pointed out that one of major reasons why patients do not seek medical care, including preventive services, is the cost barrier [3–5]. Research has further indicated that even a small cost-sharing amount was associated with reduced use of care [6]. Thus, policies expanding preventive benefits, such as more generous Medicare coverage decisions for preventive cancer screenings, bear the expectation of improved use of these services with the reduction of out-of-pocket (OOP) expenses.

When first established in 1965, Medicare paid only for services and items necessary for diagnoses or treatment of illnesses. Since the early 1990s, the Medicare Part B program has gradually extended benefits to preventive care. The Omnibus Budget Reconciliation Act of 1990 expanded coverage to include the screening mammogram and Pap smear tests for women. The colon cancer screenings were added later by the Balanced Budget Act of 1997. In general, Medicare fee-for-service (FFS) coverage of medical services requires a 20% cost-sharing amount after the yearly Medicare Part B deductible is met. These requirements initially applied to cancer screenings. But the Part B deductible was waived for most screening tests by 2007 [7], while the 20% coinsurance requirement was maintained for preventive mammogram, colonoscopy and Pap smear tests.

Despite Medicare’s efforts to promote use of screening tests by reducing patients OOP spending, studies found mixed responses to the earlier benefit expansions in Medicare [8–12]. Extensive research underscored other factors associated with screening utilization, including demographic and socioeconomic characteristics [13–17], physician recommendation [18], cost-sharing for outpatient physician visits [19], presence of supplemental insurance coverage [12, 20], patient health literacy and knowledge [21, 22], and English proficiency [23, 24].

Beginning in January 2011, the Patient Protection and Affordable Care Act (ACA) eliminated the cost-sharing requirement for a number of preventive services in Medicare FFS, including the cancer screenings recommended by the U.S. Preventive Services Task Force (USPSTF) [25]. Under the ACA, all deductible, copayment, or coinsurance was waived for preventive screenings.

The potential effects of further removing cost-sharing for preventive care in the Medicare benefit by the ACA are not clear. The ACA policy may have a very limited impact because the cost-sharing removal may not have brought a dramatic OOP cost reduction to many Medicare beneficiaries who had had supplemental coverage, including privately purchased policies, retiree coverage, or Medicaid. These supplemental benefits may have shielded beneficiaries from the OOP cost for cancer screenings prior to the onset of ACA policy. But those who are poor or not covered by supplemental insurance may respond to the extended benefits by increasing screening utilization. It can thus be expected that some Medicare beneficiaries would respond to the OOP reduction by the ACA and increase their screening utilization.

However, the potential positive effect of the ACA policy on screening test use even among the poor or those without supplemental coverage may be weakened because the OOP costs for outpatient physician visits related to the screening tests still apply. Often cancer screenings occur as referrals following visits to physicians for other medical needs. In addition, a pre-operative visit to physicians before the screening procedure (e.g. colonoscopy) may be needed, which would generate OOP expenses. As a result, the OOP cost-sharing for physician visits can be an indirect cost barrier to preventive care access [19].

In this study, we employed a comprehensive national survey of Medicare beneficiaries linked to administrative claims to examine the impacts of the ACA policy removing the cost-sharing requirements on the use of several common preventive screenings covered by Medicare. We focused on three preventive cancer screenings (mammogram, Pap smear, and colonoscopy) for which coinsurance requirements were removed in Medicare fee-for-service (FFS).

## New contribution

First, we use a comprehensive national data base that helps minimize the influence of data sources on the estimated impacts of Medicare policy. A small yet emerging literature has examined the association between the elimination of cost-sharing requirement by ACA and preventive care use in Medicare, but it has shown mixed results. One study found a small decline in mammograms among Medicare beneficiaries who experienced cost-sharing reduction [26], while others reported an increase in mammograms after removing the cost-sharing [27, 28]. Similarly, prior research indicated a rise in use of colonoscopy associated with the removal of out-of-pocket payment [29, 30], yet some found a decrease in colonoscopy use [27] by Medicare beneficiaries.

The studies using national survey data tended to find an increase in screening tests [28–30], while the studies based on claims reported an unchanged or decrease in screening tests in years after the ACA [26, 27]. Claims data and survey data differ in nature, which may contribute to the differences in findings. Claims data are derived from billings that document Medicare-covered procedures (e.g. screenings) for reimbursement to providers, but self-reported surveys collect the utilization data directly from patients and usually contain detailed information on individual socioeconomic and racial/ethnic characteristics. We bridged the gap by taking advantage of screening utilization information available in both claims and survey data.

Second, this study provides the first empirical evidence on how the cost-sharing reduction under the ACA improved the use of preventive services for patients with adverse demographic and economic characteristics in Medicare, who traditionally underuse screenings (e.g. lower income or no supplemental coverage) [14–16, 20]. As discussed above, the effects of a cost-sharing change may be larger for individuals without supplemental insurance than for those with insurance. This knowledge is currently lacking, yet it is important for policy making because these groups represent the target of the ACA reform and desirable policy effects should occur in population groups who have potential to underuse preventive services.

Third, we compare findings between outcomes measured based on the Medicare policy and those constructed following USPSTF. The existing studies have relied on recommendations from the USPSTF to measure the screening outcomes. However, there have been inconsistencies between the Medicare benefits and the USPSTF guidelines years after the implementation of the ACA. Under the ACA, Medicare is required to cover the Grade A and B screenings recommended by the 2009 USPSTF guidelines for free. Yet Medicare has always covered screenings at a broader level than the contemporaneous USPSTF guideline (Appendix 1). For example, Medicare Part B reimburses cancer screenings without an upper age cap [31] while the USPSTF recommendations vary by age (e.g. 50 to 75 years old for colonoscopy). Similarly, Medicare pays for more frequent screenings: biennial Pap smears and annual mammograms are covered by Medicare without patient cost-sharing, while USPSTF only recommends a Pap smear every 3 years and biennial mammography. Moreover, the USPSTF guidelines are continuously updated even after the ACA implementation in 2010 [32]. For example, the Pap smear recommendation was updated in 2012, from biennial to triennial tests. Because of these differences between the Medicare coverage and USPSTF guidelines, inspecting the change in cancer screening use based only on the latest USPSTF guidelines may underestimate Medicare policy effects. We thus examine measures reflecting the Medicare coverage policy, supplemented by measures following USPSTF guidelines. In addition, we investigated the changes in cancer screenings that are covered by Medicare but are not recommended by USPSTF.

Lastly, we focus on three different preventive cancer screenings (mammogram, Pap smear and colonoscopy) for which the coinsurance requirements were removed in Medicare FFS. In addition, the varying prices of these three services allow us to examine potential differences in the response to the ACA policy by screening price. Colonoscopy is one of the most expensive preventive services, and mammogram and Pap smear tests typically require low OOP costs. Given the varying costs of these screening tests, effects of the cost-sharing removal may differ by screening. For example, beneficiaries may not respond to the benefit change for Pap smear that has low costs; however, beneficiaries may be sensitive to the reduction in OOP price for colonoscopy, which has relatively high costs. We thus compare the potentially different policy effect size across three preventive screenings.

## Method

### Data

We analyzed data from community-dwelling Medicare FFS beneficiaries in the 2007–2013 Medicare Current Beneficiary Survey (MCBS), a nationally representative panel survey conducted by the Centers for Medicare and Medicaid Services. It is the most comprehensive dataset that documents demographics, socio-economic status, access to care, and insurance sources of the Medicare population. The MCBS survey data were linked to Medicare FFS claims, which contain detailed utilization data on cancer screenings.

### Study sample selection

We included beneficiaries who were continuously enrolled in the Medicare Part B program for the entire calendar year. Enrollees of Medicare Advantage plans were excluded because their claims data are not available to researchers. We excluded beneficiaries dually eligible for Medicare and Medicaid, who generally received assistance from the State Medicaid programs to meet their Medicare Part B cost-sharing requirements.

We searched the physician and hospital outpatient claims files for beneficiaries who met the study inclusion criteria to obtain diagnoses (International Classification of Diseases, Ninth Revision [ICD-9]) and procedures (Health Care Financial Agency’s common procedures coding system [HCPCS] codes & Current Procedural Terminology [CPT] codes) for billed tests. We excluded those who were likely to have diagnostic tests or have surveillance screenings because the Medicare benefit policy remained unchanged for such screening tests in this population. These included enrollees who had a claims-documented or self-reported history of total colectomy (CPT: 44150–44,153, 44,155–44,158, 44,210–44,212), mastectomy (HCPCS: 19180, 19,200, 19,220, 19,303,-19,307; ICD-9-CM: 85.24, 85.44, 85.46, 85.48, 85.41, 85.43, 85.45, 85.47) or lumpectomy (ICD-9-CM: 85.20–85.21; CPT: 19120, 19,125, 19,126), or high cervical cancer risk (V15.89).

### Cancer screening measures

Our primary outcome measures were preventive colonoscopy, mammogram, or Pap smear utilization based on extant Medicare coverage. We identified tests performed without a diagnosis or disease symptoms, based on established algorithms available from the literature [33–38]. Specifically, we examined annual mammogram use among women 40 and older, biennial Pap smear tests among all women, and use of a colonoscopy in a year among enrollees 50 and older. The unit of analysis for mammogram and colonoscopy is person-year. For biennial Pap smear, we took advantage of a rotating 4-year panel design of MCBS and limited the sample to those who were observed at least two years. A female who underwent a Pap test within the past two years was considered compliant to the biennial Pap smear screening.

While Medicare covers a colonoscopy only every ten years, we could not construct a decennial measure because of the short time frame of the panel data. We tested the sensitivity of our colonoscopy results by limiting the sample to beneficiaries for whom all four years of MCBS data were available. The outcome variable was set equal to 1 if a colonoscopy was done within the four years.

The second group of outcome measures, which supplemented the measures above, was also based on claims data and reflected alternative age spans and varied frequency specifications recommended by USPSTF. These measures included biennial mammogram use among women 50–74 years, triennial Pap smears among women 21–65 years of age, and a colonoscopy in any given year for those 50–75.

We then captured the potential variations in the policy estimates because of different data sources by constructing outcome measures based on the self-reported utilization data in the MCBS survey. Specifically, four measures were constructed based on Medicare coverage policies, including annual mammogram use among women 40 and older, biennial Pap smear tests among all women, use of a colonoscopy or sigmoidoscopy within the past 5 years among enrollees 50 and older, and whether an enrollee ever used a colonoscopy among those 50 and older. Before 2013, the MCBS survey asked about annual mammogram and Pap smear tests utilization. However, the questions changed in 2013 and only asked about people’s experiences in the past 4 years. Thus, the survey-based measures for mammogram and Pap smear screenings used data during 2007–2012.

### Statistical analysis

#### Key explanatory variable

The policy of cost-sharing removal went into effect on January 1st, 2011. This policy was represented by a 3-category ACA variable: 2007–2010 (before policy), 2011–2012 (in transition after newly implemented), and 2013 (after policy).

#### Analytical approach

The effect of the Medicare benefit expansion on the probability of receiving a cancer screening test was estimated using a logistic regression, adjusting for the complex survey design of MCBS data. The model was estimated separately for colonoscopy, mammogram, and Pap smear screening test. The standard errors were clustered at the individual respondent level to account for the fact that the same person may be observed multiple times during the survey period. The robust standard errors accounted for the autocorrelation in the panel data.

Our models included covariates that can potentially influence utilization. For individual factors, we included beneficiary income, education, age, race/ethnicity, marital status, an indicator of current smoker, and variables of trust and communication between patients and doctors. We included a binary indicator for beneficiaries under 65 who enrolled into Medicare because they received Social Security Disability Insurance (SSDI) benefits, as their health care access and utilization may be different from that of elderly beneficiaries. A binary variable indicated beneficiaries who had a supplemental private policy (e.g. Medigap) that paid for part of the Part B cost-sharing for Medicare covered services. Two market characteristics were also controlled for. We captured physician availability in a county using the number of primary-care physicians (gastroenterologists for colonoscopy) per a 100,000 population. Enrollees in managed care plans tended to use more preventive care than those in the traditional FFS program [39, 40], and spillovers from managed care may affect fee-for-service utilization and induced changes in practice patterns [41]. We controlled for this factor by including the percentage of the Medicare population enrolled in managed care within a county. These market variables were derived from the Area Health Resource Files (AHRF).

The focus of our study was to examine the effects of cancer screening coverage as a result of a major policy change. Thus, the model included a continuous time variable to control for secular trends over time, separate from policy effects. The time trends in our model reflected changes in outcomes over time that occurred regardless of the policy. For instance, Pap smear rates over time may have declined due to diminishing recommendations from doctors. Research that evaluates policy changes commonly include a linear time trend in addition to post-policy time dummy variables to control for these time trends [26]. Nonetheless, we also addressed whether or not the inclusion of a continuous time trend variable influenced the variance of our policy effects variable estimates by examining the variance inflation factor. These results are reported in Appendix 4 and indicated that adding the year trend variable did not substantially influence the variance of policy effects estimates.

The logistic regression was also separately estimated on groups with different social characteristics, such as those not having had private supplemental insurance coverage, different income and education levels, and analysis based on each race/ethnic group for each type of screening. In addition, we performed an analysis limiting the samples to those eligible for all three screenings to examine whether varying OOP prices of screening tests led to different responses from consumers.

## Results

Table 1 displays the sample characteristics. On average, approximately 38% of females aged 40 and older had a mammogram, about 7% of the sample beneficiaries over age 50 had a screening colonoscopy in any year (that is, about 70% beneficiaries would have a screening colonoscopy within a 10-year interval), and 21% of the female individuals underwent a biennial Pap test.

**Table 1:** Characteristics of Study Samples (%)

	Mammogram (women> = 40)	Biennial Pap Smear (all women)	Colonoscopy (men and women > = 50)
Average utilization based on Medicare coverage rules in claims data	38.29	20.74	6.81
Having a private supplemental insurance	84.30	85.63	82.57
Living in Metro area	69.83	70.27	69.43
Income Level: Poor	7.17	6.95	5.62
Income Level: Near Poor	28.90	26.11	23.77
Income Level: Middle	37.24	36.50	38.38
Income Level: High	26.69	30.44	32.22
Education Level: Less than high school	6.21	6.01	7.25
Education Level: High School	54.31	52.58	49.68
Education Level: College	39.48	41.41	43.07
Current Smoker	8.92	8.37	10.01
Being married	42.93	41.91	55.79
Female	…	…	53.61
Age	75.47 (10.26)	75.15 (11.67)	75.73 (8.65)
Race/Ethnicity: Hispanic	4.51	4.43	4.83
Race/Ethnicity: Non-Hispanic Black	7.49	6.47	6.66
Race/Ethnicity: Non-Hispanic Other Race	3.48	3.77	3.57
Race/Ethnicity: Non-Hispanic White	84.51	85.32	84.94
Self-reported health: Excellent	17.32	18.13	17.30
Self-reported health: Very good	32.08	33.43	31.45
Self-reported health: Good	29.57	29.98	30.41
Self-reported health: Fair	15.25	13.74	15.26
Self-reported health: Poor	5.77	4.72	5.58
Disabled	8.17	9..25	6.67
Not proficient English	1.50	1.45	1.77
Distrust Doctor	8.25	8.43	7.64
Bad Communication with Doctor	22.34	19.00	21.29
MA plan penetration	17.36 (10.37)	17.61 (10.32)	17.48 (10.33)
Primary Care Physician Number/100,000 population (Gastroenterologist Number/100,000 population for colonoscopy screening)	45.65 (24.23)	44.76 (23.81)	1.12 (1.94)
*N*	19,036	7935	38,226

The logistic regression results for policy effects for the primary outcome measures are presented in Table 2. After the cost-sharing removal, we found no statistically significant differences in a beneficiary’s probability of receiving a colonoscopy (transition period: OR = 1.08, 95% CI = 0.90–1.29; post-policy period: OR = 1.08, 95% CI = 0.83–1.42), a mammogram (transition period: OR = 1.03, 95% CI = 0.91–1.17; post-policy period: OR = 1.07, 95% CI = 0.88–1.30), or a biennial Pap smear (transition period: OR = 0.87, 95% CI = 0.69–1.09; post-policy period: OR = 0.72, 95% CI = 0.51–1.03).

**Table 2:** Effects of Cost-sharing Elimination and Covariates on Utilization. (Odds Ratio, Confidence Interval (CI), *P*-value)

	Annual Mammogram (Women > = 40)			Biennial Pap Smear (All women)			Colonoscopy (All men and women > = 50)		
	Odds Ratio	95% CI	*P*-value	Odds Ratio	95% CI	*P*-value	Odds Ratio	95% CI	*P*-value
Transition: 2011–2012	1.03	0.91–1.17	0.67	0.87	0.69–1.09	0.22	1.08	0.90–1.29	0.42
Post-policy: 2013	1.07	0.88–1.30	0.49	0.72	0.51–1.03	0.07	1.08	0.83–1.42	0.56
Continuous year indicator	0.94	0.91–0.98	< 0.001	0.97	0.89–1.05	0.45	0.97	0.92–1.02	0.20
Having a private supplemental insurance	2.12	1.84–2.43	< 0.001	1.67	1.26–2.21	< 0.001	1.92	1.64–2.26	< 0.001
Income level: Poor (Ref: High Income)	0.73	0.60–0.89	< 0.001	0.83	0.58–1.19	0.31	1.07	0.84–1.36	0.61
Income level: Near poor	0.88	0.78–1.00	0.06	0.89	0.70–1.13	0.34	0.99	0.86–1.15	0.92
Income level: Middle	0.96	0.86–1.06	0.40	1.15	0.96–1.37	0.12	1.10	0.99–1.22	0.09
Education level: Less than high school (Ref: College)	0.73	0.59–0.91	< 0.001	0.68	0.41–1.11	0.12	0.89	0.72–1.10	0.29
Education level: High school	0.93	0.84–1.02	0.14	0.78	0.65–0.93	0.01	0.93	0.84–1.02	0.13
Current smoker	0.59	0.50–0.70	< 0.001	0.75	0.55–1.02	0.06	0.78	0.66–0.93	0.01
Living in metro area	0.96	0.87–1.05	0.36	1.27	1.05–1.53	0.01	1.00	0.90–1.10	0.93
Female	–	–	–	–	–	–	0.96	0.87–1.06	0.41
Being married	1.30	1.18–1.44	< 0.001	1.48	1.23–1.77	< 0.001	1.11	1.00–1.23	0.05
Age	0.93	0.96–0.97	< 0.001	0.93	0.92–0.94	< 0.001	0.97	0.96–0.98	< 0.001
Race/Ethnicity: Hispanic (Ref: Non-Hispanic White)	0.75	0.57–0.98	0.04	01.00	0.60–1.65	0.99	0.83	0.62–1.11	0.21
Race/Ethnicity: Non-Hispanic Black	1.02	0.85–1.21	0.87	0.92	0.65–1.32	0.67	1.20	1.00–1.43	0.05
Race/Ethnicity: Non-Hispanic other race	0.83	0.64–1.08	0.16	0.84	0.56–1.27	0.41	0.78	0.56–1.08	0.13
Disabled	0.52	0.41–0.66	< 0.001	0.41	0.26–0.65	< 0.001	0.59	0.47–0.75	< 0.001
Self-reported health: Very good (Ref: Excellent)	1.13	1.01–1.26	0.04	0.97	0.80–1.17	0.74	1.08	0.94–1.23	0.27
Self-reported health: Good	1.08	0.96–1.21	0.21	0.87	0.71–1.08	0.20	1.17	1.02–1.34	0.02
Self-reported health: Fair	0.82	0.71–0.94	0.01	0.81	0.62–1.07	0.14	1.27	1.08–1.50	< 0.001
Self-reported health: Poor	0.50	0.40–0.62	< 0.001	0.61	0.41–0.91	0.02	1.16	0.90–1.49	0.24
Not proficient English	0.79	0.50–1.25	0.31	1.29	0.55–3.05	0.56	0.56	0.29–1.08	0.08
Distrust Doctor	0.93	0.78–1.10	0.38	1.06	0.77–1.47	0.72	1.12	0.95–1.33	0.17
Bad Communication with Doctor	1.07	0.96–1.20	0.22	0.87	0.69–1.10	0.23	1.14	1.02–1.28	0.03
MA plan penetration	1.00	1.00–1.01	0.22	1.00	0.99–1.01	0.62	1.00	1.00–1.01	0.33
Physician Number/100,000 population	1.00	1.00–1.00	0.01	1.00	1.00–1.00	0.99	1.00	0.98–1.03	0.74
*N*	19,036			7935			38,226		

**Fig. 1:**
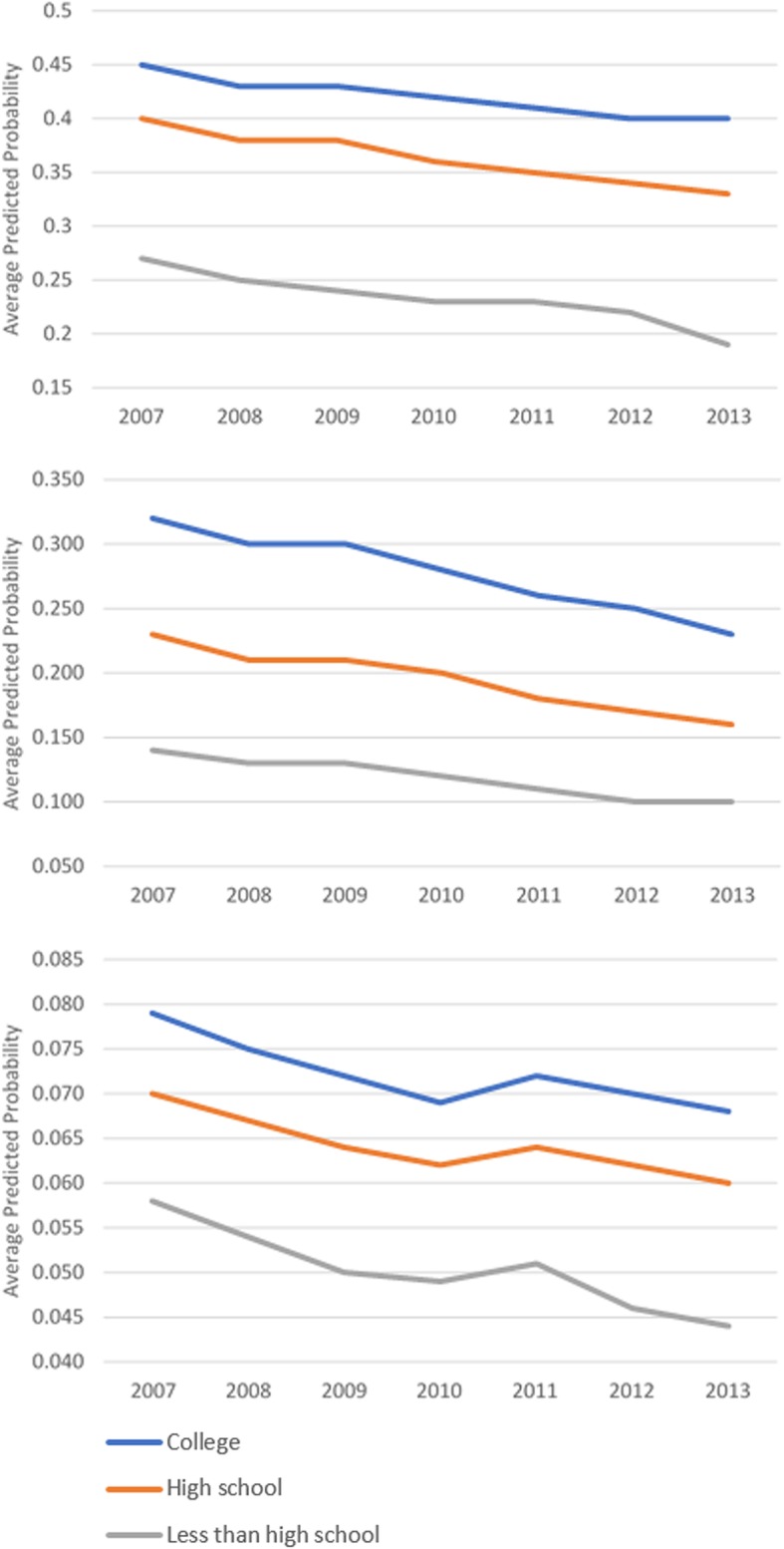
Adjusted Screening Rates by Education. **a** Mammogram, **b** Pap Test, **c** Colonoscopy

**Fig. 2:**
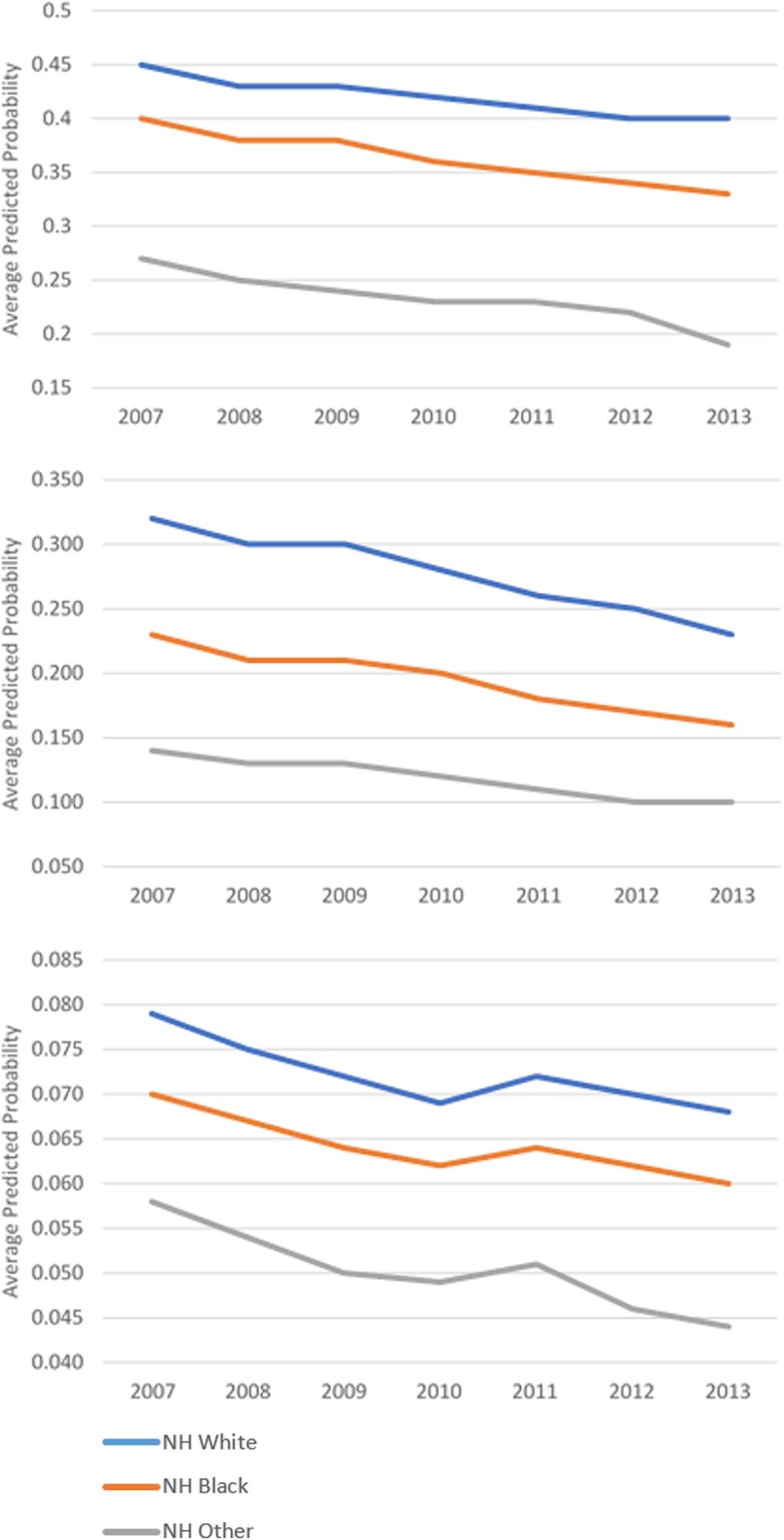
Adjusted Screening Rates by Race/Ethnicity. **a** Mammogram, **b** Pap Test, **c** Colonoscopy

**Fig. 3:**
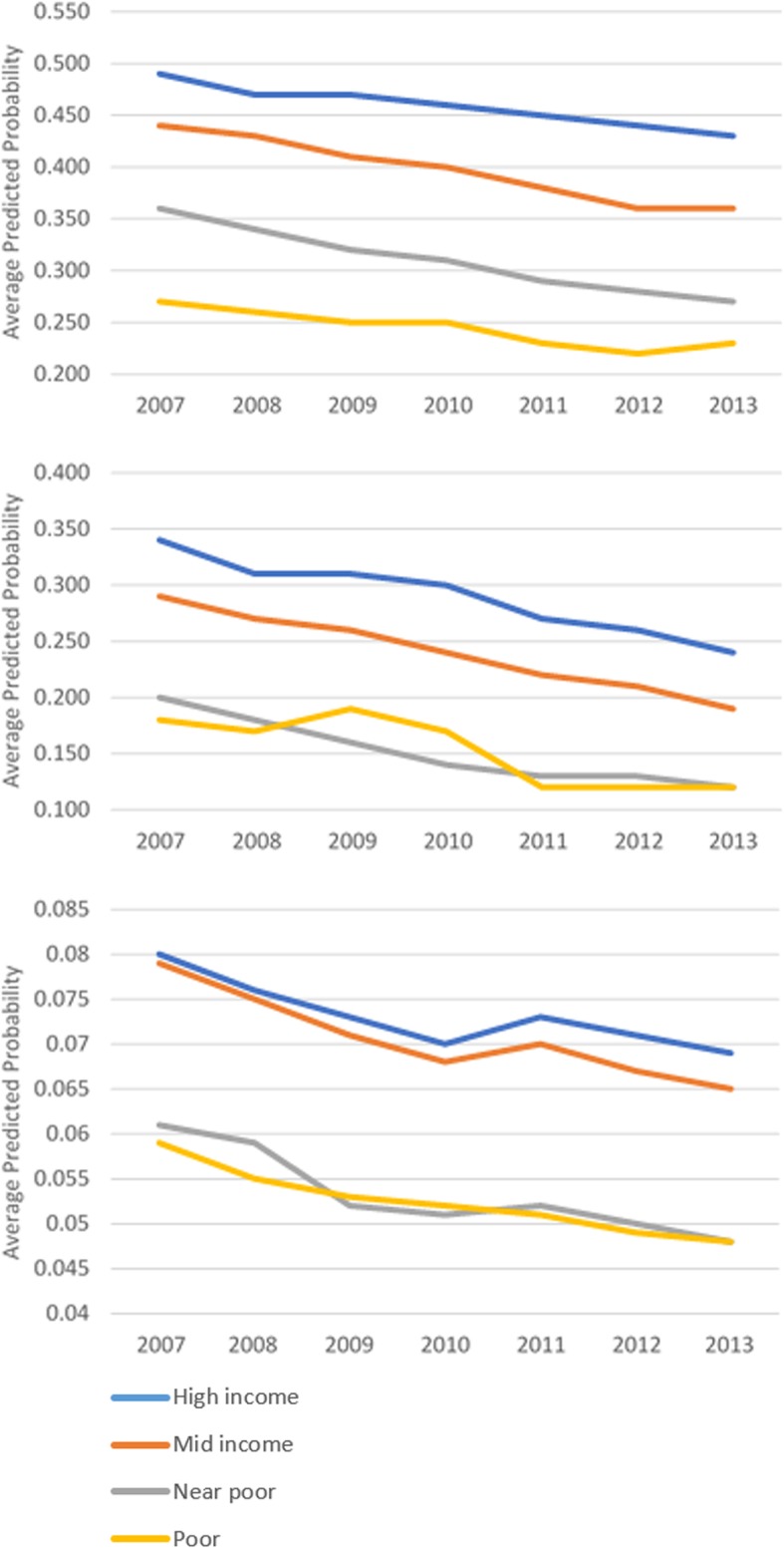
Adjusted Screening Rates by Income. **a** Mammogram, **b** Pap Test, **c** Colonoscopy

While screening utilization after the cost-sharing removal did not change, there were disparate trends in the adjusted screening rates by beneficiaries’ socioeconomic status and racial/ethnic characteristics. Over time, the predicted probabilities of all screenings slightly declined (based on first group of outcome measures), but use of screenings by those who obtained less than a high school education remained the lowest while that of beneficiaries who had college degree constantly ranked highest (Fig. 1). Both Hispanic beneficiaries and those from a racial minority group other than non-Hispanic Black had consistently low probabilities of using colonoscopy or mammogram throughout the observation period (Fig. 2). Similar disparate patterns of adjusted screening rates were observed by income groups (Fig. 3).

### Stratified analysis

Table 3 exhibited the policy effects for subgroups. Although beneficiaries who did not have private supplemental insurance were likely to be subject to cost-sharing requirements before the ACA, they did not present any statistically significant change in screening use after the elimination of the cost-sharing. Medicare enrollees who were poor were expected to be most sensitive to OOP cost change. However, after the removal of out-of-pocket cost requirements, this subgroup had a statistically significant reduction in the probability of using mammogram (Transition period: OR = 0.42, 95% CI = 0.25–0.73; Post-policy period: OR = 0.50, 95% CI = 0.21–1.21) or colonoscopy (Transition period: OR = 0.29, 95% CI = 0.12–0.68; Post-policy period: OR = 0.26, 95% CI = 0.07–0.97). While this pattern partially reflected overall decreases in screenings over time, enrollees with high- or middle-levels of income did not show any change in the utilization after the policy change.

**Table 3:** Effects of Cost-sharing Removal on Different Population Groups £. (Odds Ratio, Confidence Interval, *P*-value)

Subsample Groups		Annual Mammogram (Women > = 40)			Biennial Pap Smear (All women)			Colonoscopy (All men and women > = 50)		
		Odds Ratio	95% CI	*P*-value	Odds Ratio	95% CI	*P*-value	Odds Ratio	95% CI	*P*-value
*Not having private supplemental coverage*	Transition	0.88	0.58–1.33	0.54	0.86	0.38–1.94	0.72	0.76	0.43–1.36	0.36
	Post-Policy	0.99	0.53–1.82	0.97	0.60	0.18–1.94	0.39	0.98	0.44–2.17	0.97
		(*N* = 2993)			(*N* = 1146)			(*N* = 6669)		
*Income groups*										
High income	Transition	1.14	0.90–1.45	0.29	0.68	0.46–1.00	0.05	1.09	0.80–1.47	0.59
	Post-Policy	1.34	0.94–1.92	0.10	0.48	0.26–0.87	0.02	1.12	0.72–1.74	0.61
		(*N* = 5079)			(*N* = 2413)			(*N* = 12,315)		
Mid income	Transition	1.12	0.90–1.38	0.31	1.13	0.77–1.66	0.53	1.16	0.88–1.53	0.30
	Post-Policy	1.11	0.81–1.53	0.52	1.12	0.60–2.10	0.72	1.04	0.69–1.59	0.84
		(*N* = 7090)			(*N* = 2898)			(*N* = 14,670)		
Near poor	Transition	0.99	0.77–1.29	0.97	0.81	0.45–1.46	0.48	1.30	0.84–2.03	0.24
	Post-Policy	0.95	0.64–1.42	0.81	0.72	0.29–1.79	0.48	1.67	0.90–3.10	0.10
		(*N* = 5503)			(*N* = 2071)			(*N* = 9091)		
Poor	Transition	0.42	0.25–0.73	0.00	1.20	0.34–4.28	0.78	0.29	0.12–0.68	0.01
	Post-Policy	0.50	0.21–1.21	0.13	1.02	0.15–7.14	0.98	0.26	0.07–0.97	0.04
		(*N* = 1364)			(*N* = 553)			(*N* = 2150)		
*Education groups*										
College	Transition	1.10	0.90–1.34	0.36	0.77	0.56–1.05	0.10	1.17	0.90–1.52	0.25
	Post-Policy	1.26	0.94–1.70	0.12	0.73	0.44–1.19	0.20	1.18	0.79–1.75	0.41
		(*N* = 7517)			(*N* = 3286)			(*N* = 16,467)		
High school	Transition	0.97	0.81–1.15	0.70	1.00	0.72–1.40	1.00	1.01	0.78–1.32	0.93
	Post-Policy	0.91	0.70–1.19	0.51	0.72	0.42–1.22	0.22	1.05	0.72–1.52	0.82
		(*N* = 10,336)			(*N* = 4172)			(*N* = 18,988)		
Less than high school	Transition	1.50	0.78–2.89	0.22	1.19	0.34–4.20	0.78	0.93	0.44–1.97	0.85
	Post-Policy	1.72	0.60–4.92	0.32	1.18	0.16–8.83	0.87	0.71	0.23–2.24	0.56
		(*N* = 1183)			(*N* = 477)			(*N* = 2771)		
*Race/Ethnicity groups*										
Non-Hispanic White	Transition	1.03	0.90–1.18	0.63	0.83	0.66–1.06	0.13	1.11	0.91–1.35	0.30
	Post-Policy	1.09	0.89–1.34	0.41	0.75	0.51–1.09	0.13	1.10	0.83–1.47	0.50
		(*N* = 16,085)			(*N* = 6768)			(N = 32,464)		
Non-Hispanic Black	Transition	1.10	0.64–1.88	0.73	1.96	0.64–6.07	0.24	1.14	0.58–2.22	0.70
	Post-Policy	1.18	0.57–2.48	0.65	1.11	0.20–6.26	0.90	1.26	0.46–3.45	0.65
		(*N* = 1427)			(*N* = 514)			(*N* = 2547)		
Non-Hispanic Other	Transition	1.39	0.68–2.86	0.37	2.37	0.58–9.66	0.23	0.38	0.13–1.13	0.08
	Post-Policy	2.53	0.87–7.31	0.09	0.86	0.12–6.03	0.88	0.47	0.11–1.99	0.31
		(*N* = 665)			(*N* = 301)			(*N* = 1367)		
Hispanic	Transition	0.83	0.42–1.64	0.59	0.33	0.11–0.98	0.05	0.76	0.31–1.86	0.55
	Post-Policy	0.52	0.19–1.42	0.20	0.18	0.03–1.00	0.05	0.45	0.11–1.87	0.27
		(*N* = 859)			(*N* = 352)			(*N* = 1848)		

*£: The statistics in each cell of this table represent a separate regression analysis in similar fashion of the main analysis, controlled for covariates*

### Addition analysis

Table 4 exhibits the policy effects of USPSTF-based measures using claims data. We found null effects of the policy change on utilization of colonoscopy among enrollees 50–75 years of age (transition period: OR = 1.05, 95% CI = 0.83–1.34; post-policy period: OR = 1.01, 95% CI = 0.71–1.44), biennial mammograms by women 50–74 (transition period: OR = 1.04, 95% CI = 0.80–1.35; post-policy period: OR = 1.15, 95% CI = 0.76–1.75), and triennial Pap smear tests among women 21–65 (transition period: OR = 0.70, 95% CI = 0.21–2.37; post-policy period: OR = 0.52, 95% CI = 0.06–4.24). The findings from survey-based measures were consistent with the estimates from claims-based measures, except that we found the use of Pap smear declined after 2011 (OR = 0.71, 95% CI = 0.62–0.81) (Table 5).

**Table 4:** Effects of Medicare Policy and Covariates on Utilization (USPSTF based Measures). (Odds Ratio, Confidence Interval, P-value)

	Annual Mammogram (Women > = 40)			Biennial Pap Smear (All women)			Colonoscopy (All men and women > = 50)		
	Odds Ratio	95% Confidence Interval	P-value	Odds Ratio	95% Confidence Interval	P-value	Odds Ratio	95% Confidence Interval	P-value
Transition: 2011–2012	1.04	0.80–1.35	0.78	0.70	0.21–2.37	0.57	1.05	0.83–1.34	0.68
Post-policy: 2013	1.15	0.76–1.75	0.50	0.52	0.06–4.24	0.54	1.01	0.71–1.44	0.95
Continuous Year	0.92	0.84–1.01	0.09	1.16	0.67–2.01	0.59	1.00	0.93–1.07	0.96
Having a private supplemental insurance	2.34	1.76–3.11	< 0.001	2.63	1.01–6.84	0.05	1.95	1.59–2.40	< 0.001
Having usual source of care	–	–	–	–	–	–	–	–	–
Income level: Poor (Ref: High Income)	0.86	0.56–1.33	0.50	1.18	0.21–6.70	0.85	1.11	0.78–1.57	0.56
Income level: Near poor	1.25	0.95–1.66	0.11	1.62	0.44–5.89	0.47	1.04	0.84–1.27	0.73
Income level: Middle	1.17	0.96–1.44	0.13	3.86	1.26–11.85	0.02	1.14	0.99–1.31	0.06
Education level: Less than high school (Ref: College)	0.52	0.28–0.95	0.04	1.42	0.21–9.65	0.72	1.03	0.76–1.41	0.85
Education level: High school	0.93	0.76–1.14	0.48	1.04	0.42–2.55	0.93	0.94	0.83–1.07	0.36
Current smoker	0.68	0.51–0.91	0.01	1.15	0.51–2.60	0.74	0.76	0.62–0.94	0.01
Living in metro area	1.04	0.84–1.28	0.73	0.81	0.33–2.00	0.65	1.03	0.90–1.17	0.69
Female	–	–	–	–	–	–	1.01	0.89–1.14	0.90
Being married	1.56	1.26–1.93	< 0.001	0.59	0.23–1.54	0.28	1.14	0.99–1.31	0.06
Age	1.03	0.99–1.06	0.13	0.98	0.95–1.02	0.27	1.00	0.98–1.02	0.99
Race/Ethnicity: Hispanic (Ref: Non-Hispanic White)	0.86	0.49–1.50	0.59	3.11	0.65–14.88	0.16	0.87	0.61–1.24	0.43
Race/Ethnicity: Non-Hispanic Black	1.26	0.88–1.81	0.21	2.21	0.81–6.07	0.12	1.34	1.08–1.66	0.01
Race/Ethnicity: Non-Hispanic other race	0.70	0.44–1.10	0.12	4.21	1.34–13.23	0.01	0.82	0.54–1.24	0.34
Disabled	1.31	0.80–2.14	0.29	2.78	0.52–14.73	0.23	0.85	0.63–1.15	0.30
Self-reported health: Very good (Ref: Excellent)	1.08	0.86–1.35	0.53	5.55	0.89–34.74	0.07	1.04	0.87–1.23	0.68
Self-reported health: Good	1.01	0.79–1.29	0.93	5.60	1.02–30.79	0.05	1.14	0.95–1.36	0.16
Self-reported health: Fair	0.86	0.63–1.17	0.33	7.67	1.43–41.24	0.02	1.19	0.95–1.49	0.12
Self-reported health: Poor	0.52	0.34–0.78	< 0.001	6.60	1.00–43.64	0.05	1.14	0.82–1.58	0.43
Not proficient English	0.59	0.22–1.59	0.30	–	–	–	0.49	0.21–1.16	0.10
Distrust Doctor	0.86	0.59–1.25	0.43	1.04	0.42–2.55	0.94	1.10	0.88–1.38	0.41
Bad Communication with Doctor	1.06	0.81–1.40	0.65	0.65	0.29–1.47	0.30	1.22	1.04–1.42	0.01
MA plan penetration	0.99	0.98–1.00	0.20	1.04	1.00–1.08	0.03	1.00	1.00–1.01	0.42
Physician Number/100,000 population	0.99	0.99–1.00	< 0.001	1.00	0.99–1.02	0.75	1.01	0.98–1.04	0.62
N	3997			331			19,024

**Table 5:** Effects of Cost-sharing Elimination and Covariates on Self-Reported Utilization. (Odds Ratio, Confidence Interval (CI), *P*-value)

	Annual Mammogram (Women > = 40) (2007–2012)			Biennial Pap Smear (All women) (2007–2012)			Had Colonoscopy Within Past 5 Years (All men and women > = 50) (2007–2013)			Ever Had Colonoscopy or Sigmoidoscopy (All men and women > = 50) (2007–2013)		
	Odds Ratio	95% CI	*P*-value	Odds Ratio	95% CI	*P*-value	Odds Ratio	95% CI	*P*-value	Odds Ratio	95% CI	*P*-value
Transition: 2011–2012	0.98	0.87–1.12	0.81	0.71	0.62–0.81	< 0.001	0.91	0.81–1.02	0.11	1.02	0.90–1.16	0.76
Post-policy: 2013	–	…	N/A	–	…	N/A	0.76	0.62–0.93	0.01	0.90	0.72–1.13	0.37
Continuous year indicator	0.95	0.92–0.98	< 0.001	1.12	1.07–1.16	< 0.001	1.03	0.99–1.07	0.15	1.08	1.04–1.13	< 0.001
Having a private supplemental insurance	1.95	1.72–2.22	< 0.001	1.46	1.21–1.77	< 0.001	1.85	1.66–2.05	< 0.001	1.99	1.78–2.22	< 0.001
Income level: Poor (Ref: High Income)	0.69	0.58–0.83	< 0.001	0.67	0.52–0.87	< 0.001	0.58	0.49–0.69	< 0.001	0.53	0.45–0.64	< 0.001
Income level: Near poor	0.79	0.70–0.89	< 0.001	0.67	0.57–0.80	< 0.001	0.69	0.62–0.77	< 0.001	0.63	0.56–0.71	< 0.001
Income level: Middle	0.93	0.84–1.03	0.16	0.84	0.73–0.97	0.01	0.81	0.75–0.88	< 0.001	0.77	0.70–0.85	< 0.001
Education level: Less than high school (Ref: College)	0.71	0.59–0.86	< 0.001	0.70	0.51–0.94	0.02	0.64	0.54–0.75	< 0.001	0.51	0.43–0.61	< 0.001
Education level: High school	0.81	0.74–0.89	< 0.001	0.82	0.72–0.94	0.01	0.83	0.77–0.90	< 0.001	0.73	0.67–0.81	< 0.001
Current smoker	0.51	0.44–0.59	< 0.001	0.72	0.58–0.91	0.01	0.74	0.65–0.84	< 0.001	0.65	0.57–0.75	< 0.001
Living in metro area	1.17	1.07–1.28	< 0.001	1.32	1.15–1.51	< 0.001	1.17	1.07–1.27	< 0.001	1.22	1.11–1.34	< 0.001
Female	–	–	–	–	–	–	0.91	0.84–0.99	0.02	0.99	0.91–1.08	0.86
Being married	1.36	1.24–1.49	< 0.001	1.18	1.03–1.35	0.02	1.21	1.11–1.31	< 0.001	1.27	1.16–1.39	< 0.001
Age	0.96	0.95–0.96	< 0.001	0.95	0.95–0.96	< 0.001	0.99	0.98–0.99	< 0.001	1.00	1.00–1.01	0.17
Race/Ethnicity: Hispanic (Ref: Non-Hispanic White)	1.05	0.83–1.34	0.68	1.19	0.81–1.74	0.38	0.92	0.74–1.14	0.43	0.84	0.67–1.05	0.12
Race/Ethnicity: Non-Hispanic Black	1.36	1.16–1.61	< 0.001	1.23	0.94–1.61	0.12	1.39	1.20–1.62	< 0.001	1.20	1.02–1.41	0.03
Race/Ethnicity: Non-Hispanic other race	1.02	0.82–1.28	0.85	1.14	0.83–1.57	0.43	0.82	0.67–1.01	0.06	0.75	0.60–0.95	0.01
Disabled	0.69	0.55–0.85	< 0.001	1.00	0.73–1.38	0.98	0.67	0.56–0.80	< 0.001	0.75	0.62–0.91	< 0.001
Self-reported health: Very good	1.10	0.99–1.23	0.08	0.88	0.77–1.02	0.08	1.17	1.06–1.29	< 0.001	1.15	1.03–1.28	0.02
Self-reported health: Good	1.04	0.93–1.17	0.48	0.88	0.75–1.02	0.09	1.19	1.08–1.32	< 0.001	1.13	1.01–1.27	0.03
Self-reported health: Fair	0.74	0.65–0.85	< 0.001	0.78	0.64–0.94	0.01	1.31	1.16–1.48	< 0.001	1.33	1.16–1.52	1.16
Self-reported health: Poor	0.55	0.45–0.67	< 0.001	0.76	0.58–0.99	0.04	1.50	1.26–1.78	< 0.001	1.50	1.24–1.82	< 0.001
Not proficient in English	1.23	0.84–1.79	0.29	1.55	0.84–2.86	0.16	0.71	0.51–1.00	0.05	0.64	0.45–0.90	0.01
Distrust Doctor	1.05	0.89–1.24	0.55	0.97	0.76–1.23	0.78	1.01	0.87–1.17	0.92	1.06	0.89–1.26	0.49
Bad Communication with Doctor	1.00	0.90–1.11	1.00	0.90	0.78–1.04	0.15	1.06	0.96–1.17	0.23	0.99	0.89–1.10	0.90
MA plan penetration	1.00	1.00–1.01	0.05	1.00	0.99–1.00	0.50	1.00	1.00–1.00	0.61	1.01	1.00–1.01	0.01
Physician Number/100,000 population	1.00	1.00–1.00	0.03	1.00	1.00–1.00	0.59	1.00	0.98–1.02	0.69	0.98	0.96–1.01	0.17
N	19,638			15,172			21,593			21,458		

Turning to the analysis to examine different responses to varying OOP prices of screening tests, we first showed that the distribution of OOP spending after removing the 20% cost-sharing requirements did not have a dramatic change (Appendix 2). For example, the median OOP costs for Pap smear were close to zero before the ACA was implemented and the OOP cost for mammogram went from $9 to zero after the ACA. The reduction was larger for colonoscopy—the OOP expense decreased by one third after the policy change—yet, OOP spending for colonoscopy remained relatively high even after the ACA, likely because many screening tests started as preventive test but when other procedures were done in the process, it became a diagnostic test with a cost-sharing requirement.

Our primary analysis above did not indicate any difference in policy effects across different screenings. To further confirm, we compared the effect sizes of the policy change within females who were 50 and older, who were eligible for all screening tests (Appendix 3). Specifically, we re-estimated the probability of using a mammogram and a colonoscopy respectively, using the biennial Pap test as a comparator. Again, the ACA policy did not exhibit statistically significant effects on any utilization.

Because the recommendation for colonoscopy is every ten years, we also checked whether the result for colonoscopy was sensitive when the sample was limited to those who were observed for all four years (the longest panel available in MCBS data). Elimination of the cost-sharing did not statistically impact use of colonoscopy within a four-year period in this sub-group.

We further controlled for the role of contacts with the health care system because enrollees may have also received referrals during their office visits in addition to seeking screening tests separately. We measured the contact with the health care system by the number of ambulatory visits (physician office and outpatient services) in each year. The analyses generated results consistent with the policy effects described earlier, and we found that additional ambulatory visits were related to increased probabilities of receiving any screening test.

Finally, we conducted analysis assessing the changes in screenings performed on beneficiaries whose age was out of the Grade A or B recommendations of USPSTF guidelines based on claims records. The findings again indicated null effects from the cost-sharing removal.

## Discussion

While research has shown that the out-of-pocket expenses for preventive care lead to reduced use of services [3–5], we did not find the direct relation between the removal of the OOP cost and preventive cancer screening use in the Medicare population. The finding of a decline in Pap smear utilization observed in the survey was consistent with a general decreasing trend of Pap smear take-up rates in recent years in other national survey data [2], in accordance with a series of guideline updates to recommend less frequent Pap smear tests [32].

There are several potential reasons why the cost-sharing removal did not result in an expected increase in the preventive screening use. First, these cancer screenings were covered as Medicare benefits long before the ACA. Removal of the 20% cost-sharing requirement for these tests did not introduce large changes in out-of-pocket prices for most beneficiaries.

Second, preventive screenings, unlike treatments for medical conditions, are more at the discretion of patients. While screenings can detect cancer earlier and can potentially increase the life expectancy, many beneficiaries may forgo the preventive care because these health benefits may only occur years later. In addition, skeptical views of preventive care efficacy or limited knowledge of screenings may have played a role in beneficiaries’ decisions on receiving screening tests. Thus, free preventive care alone may not have provided incentives for Medicare enrollees to take up the care. Furthermore, because the OOP costs to regular physician visits was unchanged, many Medicare beneficiaries were not educated by providers or did not obtain referrals for screenings.

Lastly, a Medicare beneficiary could undergo sigmoidoscopy—a cheaper and less invasive procedure covered by Medicare—every 5 years instead of a colonoscopy every 10 years. Thus use of sigmoidoscopy may have responded to the benefit change. However, the sample size of people who received sigmoidoscopy was very small in our sample, thus the assessment of this test for our study was not feasible.

Our study also indicated that the cost-sharing removal did not increase screening utilization among patients with disadvantaged socioeconomic characteristics. Use of mammogram or colonoscopy even further declined among poor Medicare enrollees after the ACA. We also found disparate patterns in adjusted screening rates by socioeconomic status and race/ethnicity that persisted over time. The likelihood of obtaining screenings by those with lower income, less educated, or who were Hispanic remained consistently lower. These findings are consistent with the experience of Canada, where disparities in preventive care due to socioeconomic inequalities persisted despite the universal insurance coverage [42]. Yet these findings are concerning and suggest that offering more generous coverage may not have benefited the population groups that the policy targeted.

Future research is needed to understand whether Medicare enrollees’ OOP cost burden associated with preventive screenings were actually reduced under the ACA. Cancer screening procedures can be used to identify potential health problems (preventive) and to investigate and monitor symptoms. In fact, it is common to have some diagnostic or therapeutic procedures during a screening mammography and colonoscopy [43, 44]. When additional medical procedures (e.g. removal of polyps) are performed during the screening, the preventive screening service is instead billed as a diagnostic or therapeutic procedure in Medicare. In fact, beneficiaries observed in our sample still faced some OOP costs of colonoscopy (while reduced) after the ACA removed cost-sharing of this screening test (Appendix 2). The ACA eliminated cost-sharing requirements for preventive screenings, yet the benefit policy remained unchanged for the screening tests done for diagnostic and surveillance purposes. As a result, Medicare enrollees may still face some OOP payments related to the preventive screenings.

Our study has several limitations. First, using the administrative claims data to definitively identify preventive cancer screening can be challenging. Claims may not provide enough clinical details to distinguish between preventive screening and diagnostic tests. Nonetheless, we employed previously validated algorithms that utilized diagnostic information available in claims data to distinguish between tests ordered for diagnostic purposes from those used for screening.

A shortcoming of the MCBS data used for this study is the short panel. Because of the 4-year panel design of the MCBS, we were unable to measure screening colonoscopy over a 10-year interval. Thus, our measure of colonoscopy use reflects 10 % of the sample receiving the test in any given year. However, the clinical recommendations and Medicare coverage policy remained the same over the study time interval, and thus this measure is unlikely to produce statistically misleading results because the same measure was applied to both pre- and post-ACA periods. Further, our robustness tests on beneficiaries who were observed over four years confirmed the null policy effects. However, because we followed up only 3–4 years since the ACA policy change, some beneficiaries might not be due for another screening, making our estimates conservative. Future research may be warranted to examine colonoscopy screening to determine whether our results change when longer time intervals are examined.

## Conclusions

The elimination of cost-sharing requirements for the Medicare beneficiaries was a major policy change that sought to reduce a perceived barrier to preventive services. This study examined the impact of this policy on use of selected cancer screenings for the Medicare population. We found that removing cost-sharing requirements did not directly affect the use of mammogram, colonoscopy, or biennial Pap smear tests in Medicare.

This study provided important evidence on the impact of preventive benefit expansion in Medicare. Its finding suggests that merely making the benefit more generous by removing cost-sharing requirements may not achieve the intended goal of promoting use of preventive screenings. Policies that relocate limited resources to address social barriers may better help improve screenings among selected groups that traditionally underused preventive care.

## Appendix

**Table 6:** Medicare Cancer Screening Coverage versus USPSTF A & B Recommendations by 2015

Screenings	Medicare Coverage Policies^¶^	USPSTF Guidelines ^§^
Mammograms	Annually for female Medicare beneficiaries ≥40 (baseline at 35)	Biennial screening mammography for women 50–74 years (Grade B)
Pap smears	All female Medicare beneficiaries are eligible (Biennially for women at normal risk)	Screening for cervical cancer in women age 21–65 years with cytology (Pap smear) every 3 years (Grade A)
Colonoscopy	All men and women beginning at age 50 years, every 10 years	Adults, beginning at age 50 years and continuing until age 75 years, every 10 years (Grade A)

^¶^Medicare-Covered Preventive Services. © 2015 Medicare Rights Center. Available at https://www.ncoa.org/wp-content/uploads/medicare-preventive-services-mrc.pdf. Accessed May 14, 2019

U.S. Preventive Services Task Force Final Recommendation Statement Breast Cancer: Screening. January 2016. Available at https://www.uspreventiveservicestaskforce.org/Page/Document/RecommendationStatementFinal/breast-cancer-screening1. Accessed May 14, 2019.

U.S. Preventive Services Task Force Final Recommendation Statement Colorectal Cancer: Screening. Update of Previous USPSTF Recommendation. June 2016. Available at https://www.uspreventiveservicestaskforce.org/Page/Document/RecommendationStatementFinal/colorectal-cancer-screening2#Pod8. Accessed May 14, 2019.

U.S. Preventive Services Task Force Final Recommendation Statement Cervical Cancer: Screening. Update of Previous USPSTF Recommendation. August 2018. Available at https://www.uspreventiveservicestaskforce.org/Page/Document/RecommendationStatementFinal/cervical-cancer-screening2#Pod8. Accessed May 14, 2019.

**Table 7:** OOP Costs Associated with Screenings Before and After the ACA

	Median Before ACA	Median Before ACA among people without private supplemental coverage	Median After ACA	Median After ACA among people without private supplemental coverage
Colonoscopy	$121	$132	$81	$93
Mammogram	$9	$9	$0	$0
Pap smear	$0	$0	$0	$0

*The OOP costs are summarized based on Medicare FFS claims data*

**Table 8:** Comparative Effects of Cost-sharing Elimination and Covariates on Utilization Among Women > = 50. (Odds Ratio, Confidence Interval (CI), *P*-value)

*Comparator: A Biennial Pap Smear*						
	Annual Mammogram			Colonoscopy		
	Odds Ratio	95% CI	*P*-value	Odds Ratio	95% CI	*P*-value
Transition: 2011–2012	1.12	0.78–1.60	0.54	1.17	0.75–1.84	0.49
Post-policy: 2013	1.51	0.88–2.59	0.14	1.29	0.55–3.01	0.55
N	12,377			12,377		

**Table 9:** Variance Inflation Factors of Policy Variable Estimates

	Annual Mammogram (Women > = 40)	Biennial Pap Smear (All women)	Colonoscopy (All men and women > = 50)
Transition: 2011–2012	1.29	1.40	1.27
Post-policy: 2013	1.63	1.47	1.63
Continuous year trend	5.48	5.02	5.47

The calculation of variance inflation factors employed all other control variables in the model

## Abbreviations

ACA: The Patient Protection and Affordable Care Act
CPT: Current Procedural Terminology
FFS: Medicare fee-for-service
HCPCS: Health Care Financial Agency’s common procedures coding system
MCBS: Medicare Current Beneficiary Survey
OOP: out-of-pocket
USPSTF: U.S. Preventive Services Task Force
